# Retraction: Potential of Landsat 8 OLI for mapping and monitoring of soil salinity in an arid region: A case study in Dushak, Turkmenistan

**DOI:** 10.1371/journal.pone.0272493

**Published:** 2022-08-17

**Authors:** The PLOS ONE Editors

The *PLOS ONE* Editors retract this article [1] because it was identified as one of a series of submissions for which we have concerns about authorship, competing interests, and peer review. We regret that the issues were not addressed prior to the article’s publication.

EG, OMK, and MJA did not agree with the retraction. XW, MBudak, SAO, and MBrestic either did not respond directly or could not be reached.
